# Reach‐scale river metabolism across contrasting sub‐catchment geologies: Effect of light and hydrology

**DOI:** 10.1002/lno.10619

**Published:** 2017-07-05

**Authors:** Lorenzo Rovelli, Karl M. Attard, Andrew Binley, Catherine M. Heppell, Henrik Stahl, Mark Trimmer, Ronnie N. Glud

**Affiliations:** ^1^ Scottish Association for Marine Sciences, Scottish Marine Institute Oban United Kingdom; ^2^ Nordcee, University of Southern Denmark Odense M Denmark; ^3^ Tvärminne Zoological Station, University of Helsinki Hanko Finland; ^4^ Lancaster Environment Centre, Lancaster University Lancaster United Kingdom; ^5^ School of Geography Queen Mary University of London London United Kingdom; ^6^ School of Biological and Chemical Sciences Queen Mary University of London London United Kingdom; ^7^ Department of Ocean and Environmental Sciences Tokyo University of Marine Science and Technology Minato‐ku Tokyo Japan; ^8^ Present address: Zayed University, Dubai Academic City Dubai United Arab Emirates

## Abstract

We investigated the seasonal dynamics of in‐stream metabolism at the reach scale (∼ 150 m) of headwaters across contrasting geological sub‐catchments: clay, Greensand, and Chalk of the upper River Avon (UK). Benthic metabolic activity was quantified by aquatic eddy co‐variance while water column activity was assessed by bottle incubations. Seasonal dynamics across reaches were specific for the three types of geologies. During the spring, all reaches were net autotrophic, with rates of up to 290 mmol C m^−2^ d^−1^ in the clay reach. During the remaining seasons, the clay and Greensand reaches were net heterotrophic, with peak oxygen consumption of 206 mmol m^−2^ d^−1^ during the autumn, while the Chalk reach was net heterotrophic only in winter. Overall, the water column alone still contributed to ∼ 25% of the annual respiration and primary production in all reaches. Net ecosystem metabolism (NEM) across seasons and reaches followed a general linear relationship with increasing stream light availability. Sub‐catchment specific NEM proved to be linearly related to the local hydrological connectivity, quantified as the ratio between base flow and stream discharge, and expressed on a timescale of 9 d on average. This timescale apparently represents the average period of hydrological imprint for carbon turnover within the reaches. Combining a general light response and sub‐catchment specific base flow ratio provided a robust functional relationship for predicting NEM at the reach scale. The novel approach proposed in this study can help facilitate spatial and temporal upscaling of riverine metabolism that may be applicable to a broader spectrum of catchments.

Riverine processing of carbon has often been disregarded or considered to be of minor importance for global carbon cycling, implying that rivers merely operate as passive conduits transporting terrestrial carbon to the coasts (Schlesinger and Melack 1981). However, present estimates suggest that only half of the net terrestrial production entering riverine systems ultimately reaches the coast, while the rest is accumulated or transformed within rivers and inland waters (Cole et al. 2007). Riverine systems can therefore play an important role in processing autochthonous and allochthonous material and contribute significantly to regional and ultimately coastal biogeochemical cycles (e.g., Battin et al. 2009; Tranvik et al. 2009). The revision of the carbon cycle has highlighted the need for targeted studies on stream and river metabolism at the reach and catchment scales (Trimmer et al. 2012).

Functional trends in stream metabolism from headwaters to rivers have long been proposed (e.g., Vannote et al. 1980). Headwaters and low order (< 4) streams are expected to be predominantly net heterotrophic on an annual basis as a result of riparian vegetation and associated overgrowth, which limits in‐stream light availability and thus primary production, and as a result deliver allochthonous organic material in the form of debris further downstream. Further validations of this trend have shown that light availability, quantified as photosynthetically active radiation (PAR), represents a strong predictor of gross primary production (GPP) and net ecosystem primary production (NEP) across streams (e.g., Bott et al. 1985 Young and Huryn 1999; Mulholland et al. 2001; Bernot et al. 2010).

In addition to light, transport of allochthonous and autochthonous organic material, as facilitated by hydrology and hydrological connectivity with the land, is expected to affect the metabolic performance of streams and rivers (Heppell et al. 2017). Therefore, dynamics in stream net ecosystem metabolism (NEM) are expected to respond to such catchment‐specific functional drivers. The degree of hydrological connectivity can be expressed as the base flow index (BFI; *see* Gustard et al. 1992), which represents the proportion of river runoff derived from stored sources due to the underlying catchment permeability. BFI values range from close to 1, in permeable catchments, with a groundwater‐modulated smooth hydrograph, to ≪ 1 in flashy systems, as a result of surface runoff (e.g., during precipitation events) and a generally lower catchment permeability. Recent investigations in lowland headwaters have shown that the BFI represents a solid descriptor for the dynamics of key stream sediment processes such as nitrogen gas (N_2_) production (Lansdown et al. 2016) and nutrient dynamics in stream waters, e.g., changes in dissolved organic carbon (DOC) and nitrate (Heppell et al. 2017). Yet, the relevance of the BFI or comparable hydrological metrics as functional parameters to describe stream metabolism has not been investigated in detail.

In this study, we quantified seasonal rates of ecosystem respiration (ER), NEP, GPP, and NEM of the water column and the benthic compartment at the reach scale (∼ 150 m) in three temperate lowland sub‐catchments with contrasting geologies. The measurements are complemented by auxiliary meteorological and hydrological data to characterize the seasonal dynamics in stream metabolism across reaches with different hydrological connectivity to the land. Data are ultimately used to discuss the functional responses of the sub‐catchments to light and hydrology, their relevant timescales, and how these might be used to predict metabolic trends across time and space in contrasting riverine settings.

## Methods

### Study site

#### River Avon catchment

**Figure 1:**
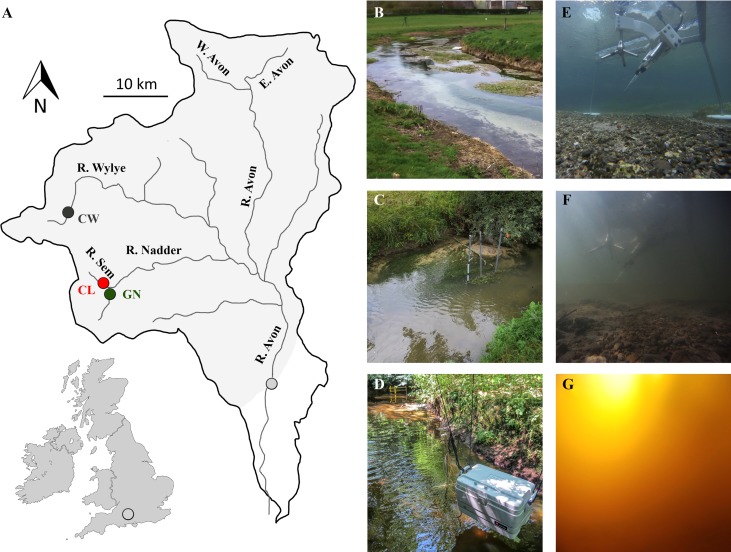
River Avon catchment. (**A**) Overview of the River Avon catchment and tributaries relevant to this study (adapted from Jarvie et al. 2005*a*). The shaded area indicates the 1393.5 km^2^ area of upper River Avon catchment, i.e., the catchment upstream of 50° 58' 28.235'' N/1° 45' 15.233'' W (light gray dot). Red dot indicates the study site within the 25.58 km^2^ catchment of CL (51° 2' 43.170'' N/2° 6' 56.315''). Gray dot indicates the study site within the 50.04 km^2^ catchment of CW (51° 9' 23.231'' N/2° 11' 24.945'' W). Green dot indicates the study site within GN, 34.16 km^2^ catchment (51° 2' 34.611'' N/2° 6' 49.500'' W). (**B–D**) Overview of the stream morphology at the study site CW (**B**), GN (**C**), and CL (**D**). (**E–G**) AEC system deployed at the CW (**E**), GN (**F**), and CL (**G**). Note that the underwater camera was mounted on the AEC frame leg at the same distance from the ADV for each of the deployments.

The study was performed within the upper reaches of the 1650 km^2^ catchment of the (Hampshire) River Avon in southern England (Fig. 1A). The catchment is mainly spring‐fed which results in relatively stable flows throughout the year, with local hydrological differences being linked to particular sub‐catchment geologies (Jarvie et al. 2005*b*). Geological data of the 1393.5 km^2^ upper catchment^1^ area showed that Chalk, Greensand, and clay represent the main catchment geologies (Allen et al. 2014), with a dominance of Chalk, which covers 82% of the upper catchment area. Greensand, which encompasses fine‐grained glauconitic sands and sandstones (*see* Bristow et al. 1999), dominates the northern (East Avon and West Avon) and western (river Nadder) parts of the catchment, and represents 12% of the total area. Only a little more than 2% of catchment area is clay, residing in the western region (river Sem) (Jarvie et al. 2005*a,b*; Allen et al. 2014). Land use within the upper catchment is largely agricultural, e.g., arable land or improved grassland (Heppell et al. 2017).

Our seasonal study focused on three ∼ 150 m long reaches of the rivers Wylye, Nadder, and Sem, with geologies dominated by Chalk, Greensand, and clay, respectively (Fig. 1B–D). The reaches were carefully selected to represent the stream morphology, riparian vegetation, and lotic vegetation of each sub‐catchment based on an extensive survey of the River Avon sub‐catchments. Each reach was investigated over a period of 3 d during each season, spanning from April 2013 to February 2014.

Throughout the manuscript, the reaches are named based on their dominant geology, as the clay river Sem (CL), the Chalk river Wylye (CW), and the Greensand river Nadder (GN).^2^ Abbreviations that are used throughout the manuscript are also listed in Table 1.

**Table 1:** List of abbreviations and symbols used in the manuscript.

Abbreviation	Definition
__b_	Benthic rate
__w_	Water column rate
__b+w_	Combined benthic and water column rate
ADV	Acoustic Doppler velocimeter
AEC	Aquatic eddy co‐variance
BFI	Base flow index
BFR	Base flow ratio
*C*	O_2_ concentration
$\bar{C}$	Time‐averaged O_2_ concentration
$C^{'}$	O_2_ concentration fluctuations
*C*_D_	Bottom drag coefficient
CL	Clay river Sem site (AS2)
CW	Chalk river Wylye site (CW2)
CTD	Conductivity‐temperature‐depth
*E*_c_	Compensation irradiance
*E*_k_	Light saturation parameter
ER	Ecosystem respiration
*F*_EC_	AEC‐based turbulent oxygen flux
GN	Greensand river Nadder site (GN1)
GPP	Gross primary production
*h*	AEC measurement height
k	von Karman constant
NEM	Net ecosystem metabolism
NEP	Net ecosystem primary production
O_2_	Dissolved oxygen
*P*	Production
*P–E*	Photosynthesis–irradiance
PAR	Photosynthetically active radiation
*P*_max_	Maximum production rate
*R*	Respiration
*U*	Flow‐velocity magnitude
u'	Longitudinal flow fluctuations
*w*	Vertical velocity
$\bar{w}$	Time‐averaged vertical velocity
w'	Vertical velocity fluctuations
*z*_0_	Sediment surface roughness parameter

### Water column incubations

Water column activity was estimated from in situ water incubations performed over 24 h. The setup consisted of a rack holding three or four sets of four 100 mL glass bottles, depending on water depth. In shallow waters (< 0.4 m), the rack held three sets: one set of clear bottles and one set of dark (light‐tight) bottles positioned near the streambed, and one set of clear bottles located near the stream surface. In deeper waters, the rack consisted of four sets, with the same configuration as in shallow waters, but with one additional set of clear bottles at mid depth (i.e., intermediate depth). Each bottle contained glass beads and was mounted so that it swayed in the flow to ensure sufficient mixing during the incubations (confirmed by adding dye to a control bottle—not shown). The concentration of O_2_ in one bottle in each of the four sets was measured continuously by O_2_ optical fibers (four channel FirestingO2; Pyro Science GmbH, Aachen, Germany) that were mounted in gas‐tight bottle caps. In shallow waters, the remaining fiber was used on a second near‐surface bottle. The average volumetric daytime and nighttime oxygen consumption or production (in μmol L^−1^ h^−1^) was quantified from the measured O_2_ concentration time series by linear regression. Nighttime was defined as the period when PAR < 2 μmol quanta m^−2^ s^−1^. In the remaining bottles (8 or 12), the net production or consumption of O_2_ during 24 h was assessed from changes in O_2_ concentration during the entire incubation period as quantified by Winkler titrations (Winkler 1888). The volumetric oxygen production or consumption rates were expressed in units of mmol m^−2^ d^−1^, by multiplying the incubation rates with average stream depth at the respective sites. The average stream depths were obtained from two seasonally determined cross‐sectional flow transects at each site (*see* Auxiliary measurements).

Light availability was monitored at each incubation site using a terrestrial PAR sensor mounted 50 cm above the water surface and an underwater PAR sensor, positioned just below the water surface; both sensor readings were logged at 30 s interval with a ULM 500 logger (Universal light meter 500; Walz GmbH, Effeltrich, Germany). Additionally, self‐contained HOBO light loggers (Onset Computer Corporation, Bourne, U.S.A.) were mounted at the height of each set of bottles to enable potential correction for vertical light attenuation within the water column.

### Benthic measurements

Benthic oxygen exchange rates were estimated using the noninvasive aquatic eddy co‐variance (AEC) technique (Berg et al. 2003). Our AEC systems were only slightly modified from the original design (Berg and Huettel 2008), and consisted of an acoustic Doppler velocimeter (ADV; Vector, Nortek A/S, Rud, Norway) and custom‐build Clark‐type O_2_ microelectrodes (Revsbech 1989) connected to submersible amplifiers (McGinnis et al. 2011) that amplified and transmitted the microelectrode signal to the ADV. The AEC system was mounted onto a small, lightweight, stainless steel tripod frame. The tripod also held an underwater camera (GoPro Hero, GoPro, San Matteo, U.S.A.), recording images every minute, to assess potential resuspension and debris that could potentially stick to the sensors and compromise the measurements.

The ADV was mounted downward‐looking, perpendicular to the riverbed surface and collected point flow measurements of each three‐dimensional (3D) velocity component at 64 Hz in a 2.16 cm^3^ sampling volume, 10–19 cm off the riverbed depending on stream morphology. The AEC frame was positioned within the main flow, with the ADV's x‐direction and O_2_ microelectrodes aligned to the flow direction, to eliminate any potential flow disruption by the frame.

One or two O_2_ microelectrodes were positioned ∼ 0.5 cm downstream of the sampling volume at an inclination of ∼ 60°. Each O_2_ microelectrode had a 90% response time < 0.5 s and stirring sensitivity < 0.5% (Gundersen et al. 1998). The potential artificial flux induced by stirring sensitivity of the microeletrodes was theoretically assessed for the prevailing flow velocities, O_2_ concentrations, bottom roughnesses and a stirring sensitivity of 0.5% using the parameterization provided by Holtappels et al. (2015). The maximum resulting artificial flux only reached 15% of the signal, at the most unfavorable conditions under high discharge. As these conditions occurred for < 10% of this study, effects of stirring sensitivity were negligible and we decided to disregard any potential influence of stirring sensitivity.

Each deployment was performed at water depths > 0.3 m and away from large upstream structures and obstacle such as, riffles, pools, bends, and fallen trees. The scheduled deployment duration was up to 3 d per site with continuous sampling. Prior to data processing, the acquired 64‐Hz datasets were averaged to 8 Hz to reduce the noise levels and to decrease the file size for more efficient data handling. During the averaging, data quality controls eliminated velocity data with beam correlations < 50% and signal‐to‐noise ratios < 10. The resulting gaps typically represented less than 1% of the dataset and were filled by linear interpolation. Averaged velocity and O_2_ time series were “despiked” with the Toolbox of Mori et al. (2007), which uses a modified 3D phase space method (Goring and Nikora 2002). The despiked velocity datasets were then rotated using double‐rotation to correct for potential instrument tilt and to eliminate the influence of vertical projections, of horizontal flow components, onto the vertical component (McGinnis et al. 2008).

The AEC‐based turbulent oxygen fluxes (in mmol m^−2^ h^−1^) were quantified from time averaged vertical velocity fluctuations ( $w^{'}$) and O_2_ concentration fluctuations ( $C^{'}$) as $F_{EC}=\bar{w'C'}$ (*see* Berg et al. 2003) using the Fortran program suite Sulfide‐Oxygen‐Heat Flux Eddy Analysis (SOHFEA) version 2.0 (available from www.dfmcginnis.com/SOHFEA; McGinnis et al. 2014). The fluctuations were obtained from measured vertical velocities (*w*) and O_2_ concentrations (*C*), and time‐averaged values ( $\bar{w}$ and $\bar{C}$) based on Reynolds decomposition as $w^{'}=w-\bar{w}$ and $C^{'}=C-\bar{C}$ via linear detrending. The window size for detrending was determined considering the effects of variable averaging time on the mean AEC flux estimates for the friction velocity (u_*_) and the oxygen fluxes (*see* Attard et al. 2014). The optimal averaging time, 5 min, included the low‐frequency turbulent flux contributions while minimizing contributions from non‐turbulent processes. Estimates of u_*_ were obtained from the Reynolds stress as $u_{*}=\sqrt{-\bar{u'w'}}$, with u' being the fluctuation along the longitudinal flow (Reidenbach et al. 2006; Inoue et al. 2011). A time‐shift correction was also applied to the datasets to align O_2_ and vertical velocity fluctuations, accounting for the O_2_ microelectrode response time, and the distance and flow‐dependent travel time between the microelectrode tip and the ADV sampling volume (Donis et al. 2015). Typical time‐shift values ranged from 0.3 s to 0.6 s.

The AEC flux footprint size, i.e., the smallest area of the streambed that contributes to 90% of the obtained flux, was estimated from the sediment surface roughness parameter (*z*_0_) and the AEC measurement height (*h*) following the parameterization by Berg et al. (2007), which assumes a well‐mixed water column, unobstructed flow, negligible influence from surface waves, and constant bottom drag coefficient (*C*_D_). The *z*_0_ was approximated as $z_{0}=h\cdot exp(-\kappa \cdot \frac{U}{u_{*}})$ with k being the von Karman constant (0.41), and U the flow‐velocity magnitude (Wüest and Lorke 2003). Averaged u_*_ values were also used to quantify *C*_D_ under law‐of‐the‐wall conditions *as* $u_{*}=U{\left(C_{D}\right)}^{1/2}$ (Wüest and Lorke 2003). The variability of *C*_D_ across the sites was primarily used to compare the reaches' hydrology, as related to riverbed morphology and associated bottom roughness.

In order to express AEC fluxes as actual benthic oxygen fluxes, the effect of transient O_2_ concentration changes in the water column between the sediment‐water interface and the AEC measurements height (*h*), termed the O_2_ storage effect, needs to be carefully weighted, as it can affect the resolved dynamics of the benthic flux estimates (*see* Holtappels et al. 2013; Rheuban et al. 2014). In this study, the O_2_ storage was quantified based on the parameterization proposed by Rheuban et al. (2014) as $F_{storage}=\int_{0}^{h}\frac{dC}{dt}dz$, with *dC*/*dt* being the hourly water column O_2_ concentration gradient. The values were compared and evaluated in relation to the measured AEC flux rates measured 10–19 cm off the bed and used to derive the actual benthic metabolism. The benthic fluxes were averaged to 1 h bins to eliminate short‐term, minute‐scale variability (e.g., Attard et al. 2014).

The light dependency of the benthic oxygen fluxes was investigated using the well‐established photosynthesis–irradiance (*P–E*) relationship of Jassby and Platt (1976), $P=P_{max}\tanh\left(E/E_{k}\right)-\left|R\right|$, with *P* (in mmol m^−2^ h^−1^) being the net production rate during the daytime, *E*_k_ (in μmol quanta m^−2^ s^−1^) the light saturation parameter, *P*_max_ the maximum production rate, and *R* the nighttime respiration rate (in mmol m^−2^ h^−1^). All fitting parameters (*P*_max_, *E*_k_, *R*) were obtained using the least squares method. In light limiting conditions, i.e., without light saturation, a linear fit was applied instead.

### Stream metabolism

Benthic and water column respiration rates, ER_b_ and ER_w_ (in mmol m^−2^ d^−1^), were calculated as the average of oxygen fluxes measured during the nighttime (in mmol m^−2^ h^−1^) and scaled to 24 h. Correspondingly, rates of net benthic and water column ecosystem production, NEP_b_ and NEP_w_, were obtained from daytime measurements. The benthic GPP rates, GPP_b_ (in mmol O_2_ m^−2^ d^−1^) were calculated as ${GPP}_{b}={NEP}_{b}+\;\left|{ER}_{b}\right|$ with |ER_b_| being the average benthic respiration rates (Lovett et al. 2006). Similarly, GPP rates in the water column, GPP_w_, were estimated as ${GPP}_{w}={NEP}_{w}+\;\left|{ER}_{b}\right|$. These procedures assume that the respiration rate is light independent (Odum 1956). It is well established, however, that benthic respiration in aquatic system during the daytime typically exceeds the nighttime rates by up to 80%, partly due to deeper daytime O_2_ penetration and partly due to larger turnover of leached labile photosynthesates (Fenchel and Glud 2000). Therefore, as in most other studies, the GPP_b_ and GPP_w_ rates represent conservative minimum estimates. The NEM (mmol m^−2^ d^−1^), representing the autotrophic–heterotrophic balance, was derived from the average respiration rate, ER, as $NEM=NEP-\;\left|ER\right|$ for both the benthic compartment (NEM_b_) and the water column (NEM_w_). Over 24 h, positive NEM values reflect net autotrophy, while negative NEM values imply net heterotrophy.

### Auxiliary measurements

#### Background measurements

Water column temperature, O_2_ concentration, and PAR near the streambed, were monitored using conductivity‐temperature‐depth (CTD) units: SBE 19 (Seabird Electronics, Washington, U.S.A.) and XR‐420 (RBR, Kanata, Canada), both equipped with Aanderaa O_2_ optode sensors (Aanderaa, Bergen, Norway) and PAR sensors (QCP‐2000 and QSP‐2200; Biospherical Instruments, San Diego, U.S.A.). The O_2_ concentration measurements were used to calibrate the AEC O_2_ microelectrodes. The PAR data were used to define nighttime periods and for *P–E* relationships. Measurements of nutrients concentrations in the water column were performed during parallel studies (e.g., Lansdown et al. 2016; Heppell et al. 2017) and are summarized in the Supporting Information Table 1. Groundwater inflow, pore‐water O_2_ concentration, and resulting groundwater oxygen fluxes were determined independently (*see* Heppell and Binley 2016*b*) and are discussed in more details elsewhere (Heppell et al. unpubl.).

#### Stream discharge

Stream discharge was estimated a under stable flow conditions over cross‐sectional flow transects and was estimated twice per site in each season, at the upstream and downstream end of the investigated reach. Flow velocity measurements were performed using an electromagnetic flow meter (Model 801; Valeport, Totnes, United Kingdom) with a vertical resolution of 0.1 m, and a horizontal resolution of 0.5 m for wide (> 3 m) and 0.25 m for narrow streams, respectively. The measurements were averaged over 15 s (*n* = 15) and the discharge was estimated by integrating the measurements over the cross‐sectional area. The transects were also used to obtain stream widths and to quantify average water depths. Complementary continuous discharge monitoring was performed in the vicinity of our sampling locations over approximately 2 yr from June 2013 to June 2016 (*see* Heppell and Binley 2016*a* 3; Heppell et al. 2017).

## Results

The metadata are summarized in Table 2 and cover 453 h of AEC measurements and 578 h of CTD measurements. The data were collected during four field campaigns covering spring (22 April 2013–06 May 2013), summer (30 July 2013–13 August 2013), autumn (29 October 2013–09 November 2013), and winter (27 January 2014–05 February 2014). Stream widths ranged from 2.5 m to 7.0 m, while average water depths ranged from 35 cm to 76 cm. Throughout the study, the average daily stream temperature ranged from 5.8°C in the winter to 18.5°C in the summer with the largest daily excursion, 6.2°C, being observed during the spring campaign. Average temperatures during each campaign (Table 2) agreed well with monitoring data (Heppell et al. 2017) and differed by less than 1–8% from monthly averages of the respective seasons. Average stream flow velocity varied from 2.3 cm s^−1^ to 46.1 cm s^−1^ depending on the site and season, but remained fairly constant within the respective 3‐d campaigns. Stream discharge ranged from 0.006 m^3^ s^−1^ to 1.123 m^3^ s^−1^ (Table 2). Mean discharge during each campaign varied by less than 12% from seasonal monthly discharges during the campaigns (*see* Heppell et al. 2017), indicating that the hydrological conditions during the campaigns were representative of each season. The highest flow velocities and discharges were observed during the 2014 winter as the result of an extreme flood (< 1 in 100 yr). Average u_*_ values ranged from 0.2 cm s^−1^ to 2.5 cm s^−1^ and revealed a consistent cross‐seasonal linear relationship to the average stream flow velocities. The bottom drag coefficient, *C*_D_, of the upper catchment amounted to 0.0033, on average, (*R* ^2^ = 0.89; Supporting Information Fig. 1) and the average sediment roughness parameter, *z*_0_, ranged from 1.0 ± 0.6 mm at CL to 0.8 ± 1.1 mm at CW, to 0.5 ± 0.4 mm at GN. Daily PAR values, at the streambed, ranged from < 5 mol quanta m^−2^ d^−1^ in the winter to 28.6 mol quanta m^−2^ d^−1^ during the summer. Nitrate and phosphate concentrations in the water column at each site exhibited seasonal variability but were overall high, with minimum values of 59 μmol L^−1^ and 0.5 μmol L^−1^, respectively (Supporting Information Table 1).

**Table 2:** Characteristics of the observational period for each field campaign at the CL, CW, and GN. Values for the respective parameters are reported as mean ± SD and as a minimum–maximum value range within brackets. Average values and range for temperature and O_2_ were obtained from CTD measurements, the average flow from AEC measurements. Stream width, depth, and discharge were obtained from cross‐sectional flow transects which were performed at the upstream and downstream end of the reach during periods with stable hydrograph (i.e., no apparent change in flow or water depth over at least 24 h). The sampling duration is defined as the number of hours of continuous measurements since the sampling started.

Campaign	Site	Starting date	Duration (h)	Temperature (°C)	O_2_ (% saturation)	O_2_ (μmol L^−1^)	Width (m)	Depth (m)	Flow (cm s^−1^)	Discharge (m^3^ s^−1^)
Spring	CL	01 May 2013 14:48:47	50.7	10.1 ± 1.0 (8.2–13.7)	95.0 ± 13.4 (77.3–118.6)	333.3 ± 41.5 (278.5–406.0)	3.0–3.5	0.39 ± 0.09	4.3 ± 0.1	0.057
	CW	22 Apr 2013 12:27:14	54.4	11.2 ± 1.6 (9.3–15.0)	101.0 ± 21.2 (77.8–137.1)	343.7 ± 60.5 (274.8–443.6)	5.0–6.0	0.35 ± 0.12	19.9 ± 0.5	0.204
	GN	04 May 2013 14:40:10	52.1	12.3 ± 1.6 (9.5–15.7)	107.3 ± 20.6 (83.9–139.9)	357.0 ± 62.0 (277.6–450.9)	3.0–3.5	0.40 ± 0.11	27.7 ± 0.1	0.312
Summer	CL	30 Jul 2013 14:57:03	49.2	17.1 ± 0.4 (16.6–18.5)	63.0 ± 2.0 (59.3–68.42)	190.2 ± 5.6 (179.6–205.8)	3.0–3.5	0.39 ± 0.09	2.3 ± 0.1	0.006
	CW	11 Aug 2013 15:58:22	50.5	13.3 ± 1.6 (10.7–16.6)	78.9 ± 27.7 (49.0–130.3)	257.2 ± 86.1 (156.6–414.2)	2.5–5.0	0.37 ± 0.06	7.6 ± 1.4	0.035
	GN	02 Aug 2013 11:41:51	54.2	16.0 ± 0.8 (14.9–17.4)	78.9 ± 5.4 (71.6–87.9)	243.1 ± 16.8 (217.5–273.6)	3.5–4.0	0.37 ± 0.14	23.0 ± 0.7	0.232
Autumn	CL	07 Nov 2013 15:45:00	49.1	9.9 ± 0.7 (8.9–11.3)	75.4 ± 0.4 (74.6–79.3)	266.8 ± 5.4 (258.4–287.1)	3.0–3.5	0.79 ± 0.24	14.3 ±0.6	0.426
	CW	29 Oct 2013 10:52:01	53.8	9.7 ± 1.0 (7.5–10.9)	78.1 ± 7.9 (67.1–92.0)	277.8 ± 28.8 (234.2–330.0)	2.5–6.5	0.38 ± 0.15	12.3 ± 0.2	0.030
	GN	04 Nov 2013 13:10:30	51.0	9.9 ± 0.5 (8.9–11.22)	74.0 ± 1.2 (70.9–76.0)	261.5 ± 6.0 (246.3–272.7)	4.0–4.5	0.49 ± 0.08	22.6 ± 3.1	0.422
Winter	CL	04 Feb 2014 13:17:54	9.2	6.0 ± 0.1 (5.8–6.1)	76.4 ± 0.5 (74.1–77.6)	296.9 ± 2.6 (287.2–302.8)	3.5–4.0	0.76 ± 0.14	25.7 ± 1.6	0.630
	CW	27 Jan 2014 17:15:42	54.6	8.5 ± 0.3 (6.8–9.1)	80.3 ± 2.6 (77.6–90.1)	295.0 ± 12.7 (278.6–363.7)	6.0–7.0	0.55 ± 0.14	46.1 ± 1.1	1.123
	GN	30 Jan 2014 16:46:12	47.9	7.0 ± 0.3 (6.4–7.6)	85.7 ± 2.1 (81.6–88.9)	325.0 ± 9.6 (307.9–337.5)	3.5–4.5	0.52 ± 0.16	37.9 ± 0.7	0.537

During the spring and summer, the O_2_ dynamics within the water column followed a typical diel pattern; undersaturation (down to 49%) during the nighttime and oversaturation (up to 140%) during the daytime (Table 2). During the spring, changes in diel O_2_ concentration were largest at the GN and CW reaches (169–173 μmol L^−1^ range), which also recorded the highest levels of oversaturation (Table 2). In contrast, the smallest diel O_2_ concentration amplitude and lowest peak oversaturation (119%) were observed in the more turbid clay reach (CL). Nighttime O_2_ concentrations during the spring were comparable between sites (∼ 277 μmol L^−1^), with O_2_ saturation ranging from 77% to 84%, mainly depending on nighttime temperature differences (Table 2). During the summer, the CW reach showed daytime oversaturation (up to 130%) and well‐defined diel O_2_ fluctuations (257.6 μmol L^−1^ in amplitude). In contrast, the CL and GN reaches were continuously under‐saturated with respect to O_2_, but still exhibited daily O_2_ oscillations ranging from ∼ 26 μmol L^−1^ at CL to ∼ 60 μmol L^−1^ at the GN reach (Table 2). During the autumn and winter, the water was undersaturated (67–92%) at all sites; diel O_2_ variations were reduced at CW (85–96 μmol L^−1^) and marginal at the CL and GN reaches (11–30 μmol L^−1^).

### Water column metabolism

**Figure 2:**
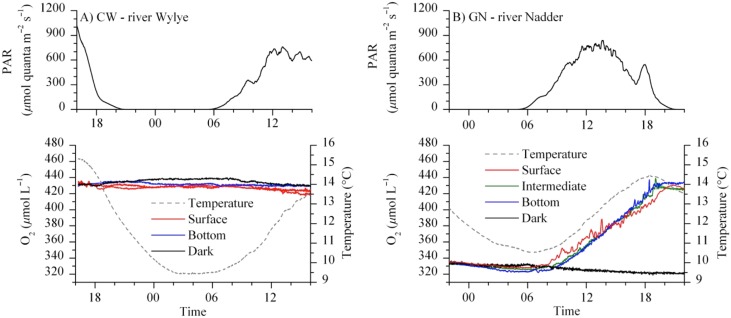
Water column incubation at CW (CK, **A**) and GN (**B**) during the spring. The top panels show the hourly‐averaged PAR measurements (in μmol quanta m^−2^ s^−1^) just below the water surface. The bottom panels display the optical‐fiber‐based O_2_ concentrations within the clear bottle O_2_ at the river surface (red lines), bottom (blue lines) and midstream depth (intermediate; green lines), as well as within the dark bottle (black lines); the water temperature timeseries is also shown (gray dashed lines). Note the evident water column activity at GN in contrast to CW, where water column activity was insignificant during the 24 h incubation period.

Oxygen production in the water column, dark respiration, and NEM_w_ rates were obtained at each site during each campaign except during the extreme 2014 winter flooding at the CL reach. Suspended sediment concentrations decreased across the reaches, from CL to GN to CW (Heppell and Binley 2016*a*) leading to a clear gradient in water column turbidity (Fig. 1). Within the respective reaches, however, O_2_ rates for the surface, midstream and bottom bottle incubations at each reach were not significantly different (e.g., Fig. 2). This indicated that depth attenuation in light was negligible at all the targeted reaches (Table 2). Therefore, the individual rates were averaged and presented as daytime (clear bottles) and nighttime (dark bottles) values, respectively (Table 3). Metabolic activity in the water column was highly variable between sites and seasons. As an example, data recorded at the CW and GN reaches during the spring campaign reflected a distinct production of O_2_ in the water column at the GN reach (average 9.0 μmol L^−1^ h^−1^), but negligible activity in the water column at the CW reach (average < 0.1 μmol L^−1^ h^−1^; Fig. 2). At the CL reach, we observed strongly elevated production rates of up to 43.2 μmol L^−1^ h^−1^, whereas dark bottle respiration rates were comparable with values from GN (1.0–1.6 μmol L^−1^ h^−1^; Table 3). Contrastingly, during the summer, the water column production was elevated at the CW reach (5.3 μmol L^−1^ h^−1^), marginal at the CL reach (0.1 μmol L^−1^ h^−1^) and negligible at the GN reach (Table 3). Respiration rates at the CL reach were higher than during the summer campaign than during the spring, while the observed rates at the other two sites were comparable between spring and summer. During the autumn and winter campaigns, no net production was observed at any time at any of the three sites. Daytime rates were negative, ranging from −1.6 μmol L^−1^ h^−1^ during the autumn to −0.3 μmol L^−1^ h^−1^ during the winter (Table 3). Across the year, GPP_w_ rates ranged from < 0.1 mmol m^−2^ d^−1^ to 268.1 mmol m^−2^ d^−1^, on average, with the maximum rates being observed at the CL reach during the spring. Average ER_w_ rates ranged from < 0.1 mmol m^−2^ d^−1^ to 26.2 mmol m^−2^ d^−1^, with the latter observed at the GN reach during the autumn (Supporting Information Fig. 2). NEM_w_ at the GN reach was positive during the spring while the remaining campaigns reflected a net heterotrophic water column (Table 3). At the CL reach, the water column was net heterotrophic from summer to winter. During the spring campaign, the water column was, however, strongly autotrophic following a peak in primary production (Table 3). O_2_ time series measurements during the campaign (this study) and concurrent monitoring of DOC concentrations (Heppell et al. 2017) indicated that the production peak was short‐lived (∼ 1 week). For extrapolation, we assumed that the average NEM_w_ rates at the CL reach for the remaining period of spring (i.e., 11 weeks) were equal to the summer rates, which resulted in an integrated whole‐spring NEM_w_ of ∼ 14.2 mmol m^−2^ d^−1^ (Table 3). At the CW reach, NEM_w_ was positive during the summer, while net heterotrophy was observed during the autumn and winter (Table 3).

**Table 3:** Seasonal water column volumetric O_2_ rates for the CL, CW, and GN. Values for day and night O_2_ rates are reported as bottle‐averaged, i.e., depth‐averaged values. Daytime rates were obtained from the average daytime production rates in clear bottles (*n* = 3). Night time rates were obtained by averaging respiration rates in the dark bottles during the daytime and at night (*n* = 2). The rates were obtained from linear regressions of the respective O_2_ time series (*R* ^2^ > 0.8).

Campaign	Site	Day (*n* = 3) (*μ*mol L^−1^ h^−1^)	Night (*n* = 2) (*μ*mol L^−1^ h^−1^)	Total PAR (mol quanta m^−2^ d^−1^)	Daylight day/night (h)	ER_w_ a (mmol m^−2^ d^−1^)	GPP_w_ a (mmol m^−2^ d^−1^)	NEM_w_ a, b (mmol m^−2^ d^−1^)
Spring	CL	43.2	−1.6	6.4	15.3/8.7	14.9	268.1	253.2/14.2^c^
	CW	<0.1	<0.1	20.2	15.2/8.8	<0.1	<0.1	<0.1
	GN	9.0	−1.0	18.4	16.4/7.6	12.1	84.5	65.0
Summer	CL	0.1	−3.6	0.5	15.4/8.6	21.4	13.9	−7.5
	CW	5.3	−1.9	21.4	15.2/8.8	13.7	32.7	19.0
	GN	<0.1	−1.2	12.1	15.9/8.1	12.6	<0.1	−12.6
Autumn	CL	−0.9	−1.3	0.3	8.9/15.1	9.1	<0.1	−8.1
	CW	−0.3	−0.3	6.0	10.9/13.1	3.8	<0.1	−3.8
	GN	−1.2	−1.6	2.0	10.4/13. 6	26.2	<0.1	−23.5
Winter	CL	n.d.	n.d.	n.d.	n.d.	n.d.	n.d.	−8.1^d^
	CW	<0.1	−0.3	0.6	9.9/14.2	2.5	<0.1	−2.5
	GN	−0.3	−0.3	0.6	8.8/15.2	4.9	<0.1	−5.1

a Water‐column based ecosystem respiration (ER_w_), GPP and (GPP_w_), and net water column metabolism (NEM_w_) were expressed as a flux (mmol m^−2^ d^−1^) by multiplying the volumetric rates (mmol m^−3^ d^−1^) by the average stream depth (Table 2).

b Net water column metabolism, NEM_w_, for sites with no detectable daytime rates, was computed by multiplying the hourly nighttime‐dark average rates by 24.

c Whole spring estimates of NEM_w_ assuming 1 week of peak primary production and the remaining 11 weeks with NEM_w_ = summer NEM_b_.

d Winter NEM_w_ rate is assumed equal to the autumn NEM_w_ rate.

### Benthic metabolism

Benthic oxygen fluxes based on AEC measurements were obtained at each site for each season, although higher discharge and flooding during the autumn and winter campaigns restricted access to the streams at some sites. The site‐specific AEC footprint area, ranged between 22–181 m in length and 0.5–1.5 m in width, resulting in an average area of ∼ 64 m^2^ at the CK and GN reaches, and ∼ 53 m^2^ at the CL reach. During the 3‐d measurements at each site, water column O_2_ concentration changed by up to 41.8 μmol L^−1^ h^−1^ (Table 2), most notably at dusk and dawn. Under these conditions, the O_2_ storage‐driven “bias” in the AEC fluxes amounted to a maximum of 6.2 mmol m^−2^ h^−1^ and was accounted for (*see* Methods).

**Figure 3:**
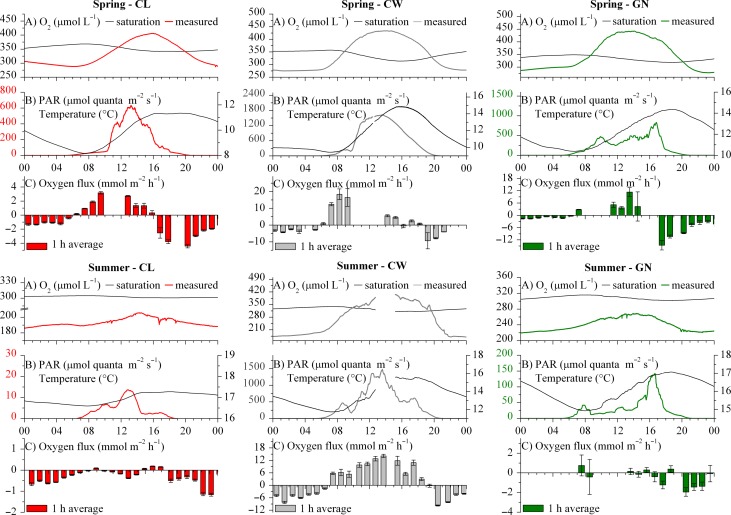
Typical benthic oxygen fluxes and auxiliary time series data during the spring (top boxes) and summer (bottom boxes) at the CL (left), CW (CK, center), and GN (right). Each box represents a 24 h selection of (**A**) in situ near‐riverbed absolute O_2_ concentration (red, gray, or green lines) and O_2_ concentration at saturation (black lines), (**B**) streambed PAR in μmol quanta m^−2^ s^−1^ (red, gray, or green lines) and water column temperature (black line), and (**C**) turbulent benthic oxygen fluxes with standard error. AEC‐based fluxes are expressed as benthic rates by inclusion of the O_2_ storage term (*see* Methods). The same figure with AEC oxygen fluxes without the O_2_ storage correction is available as Supporting Information Fig. 3. Time series of PAR were averaged to 1 h to highlight the dominant trends.

Typical 24 h datasets of corrected benthic oxygen fluxes, from the spring and summer campaigns, are depicted in Fig. 3. The benthic oxygen flux dynamics reflected the light availability at all sites during the spring, most noticeably at the CW and GN reaches (Fig. 3). Daytime benthic fluxes were positive (up to 26.3 mmol m^−2^ h^−1^), while nighttime fluxes were negative and often associated with a clear decrease in ambient water O_2_ concentration. Overall, oxygen fluxes at the CW reach during the spring ranged from −9.4 mmol m^−2^ h^−1^ to 18.4 mmol m^−2^ h^−1^. Lower positive oxygen fluxes were observed at the GN reach (−14.6 mmol m^−2^ h^−1^ to 11.3 mmol m^−2^ h^−1^), while the lowest oxygen fluxes were observed at the CL reach (Fig. 3). Oxygen flux at the turbid CL reach, also showed little daily variation and a more dampened light response compared to the other two sites (Fig. 3), with nighttime fluxes around −1.6 mmol m^−2^ h^−1^ and with only sporadically positive daytime fluxes (up to 3.2 mmol m^−2^ h^−1^). Integrated on a daily basis, the net metabolism remained heterotrophic (Table 4).

**Table 4:** Seasonal benthic oxygen fluxes and associated benthic metabolism for CL, CW, and GN. Day and night oxygen fluxes are reported as whole‐campaign averages as a mean ± SE (*n*), with *n* indicating the number of hourly averaged values, and include the O_2_ storage correction to express the AEC fluxes as benthic fluxes.^a^

Campaign	Site	Day flux (mmol m^−2^ h^−1^)	Night flux (mmol m^−2^ h^−1^)	Daylight day/night (h)	Total PAR (mol quanta m^−2^ d^−1^)	ER_b_ (mmol m^−2^ d^−1^)	GPP_b_ (mmol m^−2^ d^−1^)	NEM_b_ (mmol m^−2^ d^−1^)
Spring	CL	−0.2 ± 0.1 (30)	−1.6 ± 0.0 (36)	15.3/8.7	10.8	38.9	21.8	−17.3
	CW	3.4 ± 0.9 (21)	−4.4 ± 0.3 (10)	14.8/9.2	27.5	104.9	115.7	10.8
	GN	0.6 ± 0.5 (14)	−3.3 ± 0.3 (20)	15.8/8.2	21.8	79.9	62.0	−17.9
Summer	CL	−0.4 ± 0.1 (26)	−0.4 ± 0.0 (19)	15.5/8.5	0.3	8.6	<0.1	−9.1
	CW	8.6 ± 1.1 (29)	−4.4 ± 0.3 (19)	15.1/8.9	28.6	105.1	196.0	90.9
	GN	0.1 ± 0.2 (25)	−1.8 ± 0.4 (12)	15.9/8.1	1.7	42.5	27.6	−14.9
Autumn	CL	−0.6 ± 0.4 (9)	−6.0 ± 0.8 (19)	9.9/14.1	0.3	143.6	<0.1	−89.8
	CW	5.0 ± 0.8 (22)	−2.2 ± 0.1 (28)	11.0/13.0	8.6	52.7	79.4	26.8
	GN	2.2 ± 1.1 (3)	−7.5 ± 3.8 (5)	10.3/13.7	0.9	179.4	<0.1	−79.1
Winter	CL	−2.7 ± 0.7 (12)	−1.5 ± 0.5 (19)	10.0/14.0	0.5	36.6	<0.1	−48.1
	CW	−0.8 ± 0.5 (13)	−0.4 ± 0.2 (29)	9.8/14.2	4.7	8.6	<0.1	−13.4
	GN	−5.4 ± 1.5 (11)	−0.7 ± 0.4 (14)	10.2/13.8	0.7	16.9	<0.1	−65.5

a Integrated rates of GPP_b_ and ER_b_ based on storage‐corrected benthic fluxes were 43% and 27% higher than from uncorrected AEC fluxes. Corrected average NEM_b_ rates were about 34% higher.

Light availability at the CW reach during the summer was comparable to conditions during the spring, and the diurnal light dynamics were clearly reflected in the benthic oxygen fluxes (Fig. 3), which ranged from −9.4 mmol m^−2^ h^−1^ at nighttime to 26.3 mmol m^−2^ h^−1^ at daytime. However, at the GN and CL reaches, extensive bank vegetation and overgrowth by trees during the summer limited light availability at the streambed; the oxygen fluxes at the GN reach were relatively small ranging between −1.3 mmol m^−2^ h^−1^ and 0.2 mmol m^−2^ h^−1^, while fluxes at the CL reach were predominantly negative throughout the day (−1.2 mmol m^−2^ h^−1^ to 0.2 mmol m^−2^ h^−1^ range).

At the CW reach, the average daytime oxygen fluxes during the autumn were still positive, on average 5.0 mmol m^−2^ h^−1^, and the magnitude exceeded the oxygen consumption rates measured during the nighttime (Table 4). Autumn nighttime oxygen fluxes at the CL and GN reaches reached values as low as −6.0 mmol m^−2^ h^−1^ and −7.5 mmol m^−2^ h^−1^, respectively. During the winter, all sites constantly exhibited negative oxygen fluxes, and oxygen fluxes at CW were on average smaller than those observed at the CL and GN reaches (Table 4).

**Figure 4:**
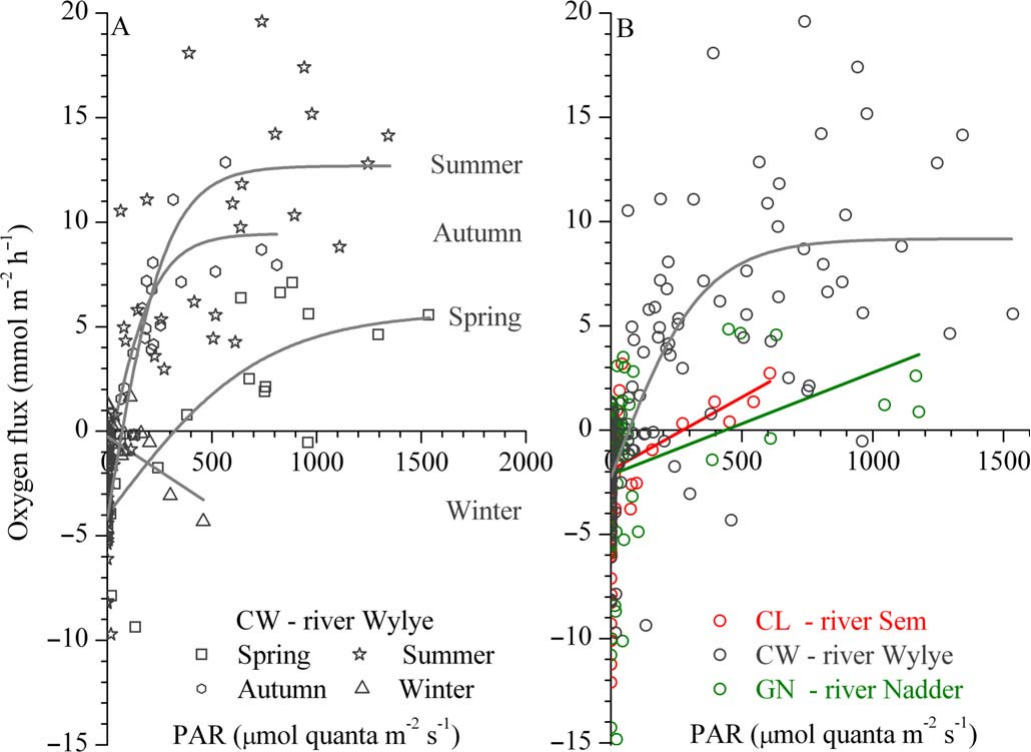
Production–Irradiance (*P–E*) relationships based on hourly averaged of AEC‐based benthic metabolism and PAR (in μmol quanta m^−2^ s^−1^). (**A**) Seasonal *P–E* relationships at the CW. Note that during the winter oxygen uptake rates were mostly correlated with temperature and thus higher during daytime. Averaged *R* ^2^ ranged from 0.69 during the spring to 0.93 during the autumn. (**B**) Integrated annual *P–E* relationships for each reach based on the available benthic oxygen fluxes.

The benthic communities responded strongly to light availability at the CW reach. This was clearly reflected by the fitted *P–E* relationships for the respective seasons (Fig. 4A). The *P–E* relationships expressed light‐saturation, but no evidence of photo‐inhibition (Fig. 4A). Fitted respiration rates during darkness were about 4 mmol m^−2^ h^−1^ during the spring and summer and reduced to 2.2 mmol m^−2^ h^−1^ during the autumn. Conversely, *P*_max_ was highest during the summer (16.9 mmol m^−2^ h^−1^) and lowest during the spring, despite very similar total daily‐integrated PAR values (∼ 28 mol quanta m^−2^ d^−1^). Both *E*_k_ and compensation irradiance (*E*_c_, i.e., PAR at *P* = 0) were highest during the spring and lowest during the autumn, but the absolute values shifted markedly between spring and summer, suggesting a functional or absolute change in the photosynthetic biomass (Fig. 4A). During the winter, no significant light response was observed, reflecting an apparent lack of primary production. The annual *P–E* curve, based on all observations, although scattered (*R* ^2^ = 0.58), reflected a clear light response across seasons (Fig. 4B). Average annual values for *P*_max_ and *R* were 11.4 μmol m^−2^ h^−1^ and 2.2 μmol m^−2^ h^−1^, respectively.

At the GN reach, spring *P*_max_ was up to 5 mmol m^−2^ h^−1^ with an *R* value of 3 mmol m^−2^ h^−1^. Compared to the CW reach, *E*_k_ and *E*_c_ were 78–86% lower. During the summer, riparian overgrowth reduced light availability in the river to 0.3–1.7 mol quanta m^−2^ d^−1^ at both the GN and CL reaches (Table 4) and presumably dampened light responses. Therefore, annual *P–E* curves at those sites did not exhibit light saturation and were overall characterized by a narrower range of production rates than seen at the CW reach (Fig. 4B).

**Figure 5:**
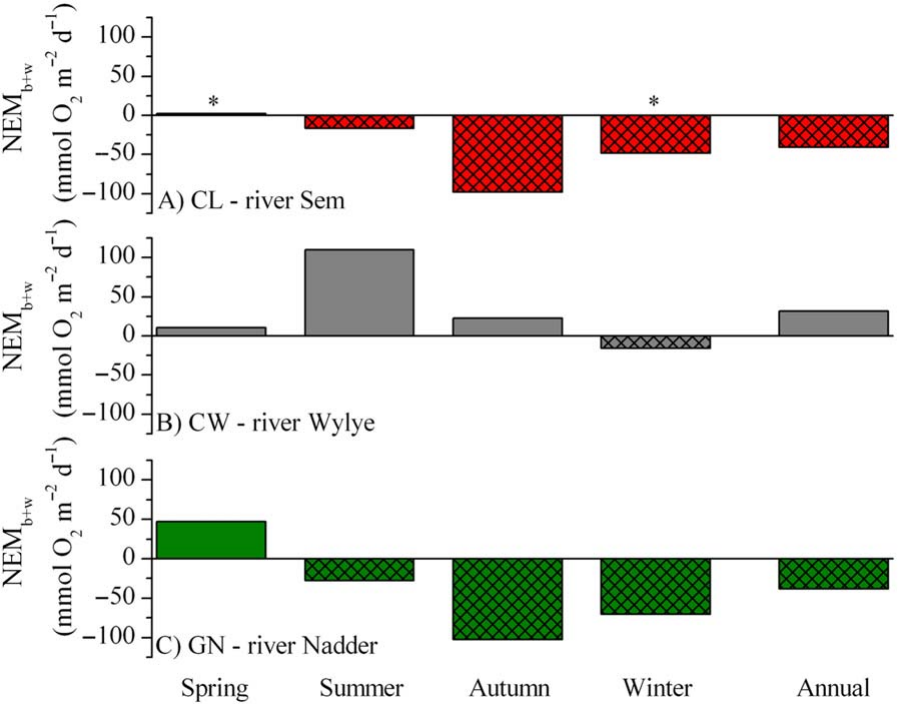
Stream net ecosystem metabolism (NEM_b+w_). The rates for the CL (panel **A**), CW (panel **B**), and GN (panel **C**) represent the combined benthic and water column contributions (NEM_b+w_) for each season and the resulting annual average (in mmol m^−2^ d^−1^). *, seasonal NEM_b+w_ at CL for the whole‐spring period was calculated based on a spring production peak of 1 week duration (*see* Results). The winter NEM_w_ rates were assumed to be equal to the autumn NEM_w_ (*see* Table 3).

The average GPP_b_ at the three sites ranged from < 0.1 mmol m^−2^ d^−1^ to up to 196.0 mmol m^−2^ d^−1^ (Supporting Information Fig. 2), with the latter observed during the summer campaign at the CW reach (Table 4). At the CW reach, ER_b_ ranged from 8.6 mmol m^−2^ d^−1^ to 105.1 mmol m^−2^ d^−1^ while NEM_b_ rates remained positive from spring to autumn, ranging from 10.8 mmol m^−2^ d^−1^ to 90.9 mmol m^−2^ d^−1^, and only reached a negative minimum of −13.4 during the winter (Fig. 5). At the CL and GN reaches, the ER_b_ rates exceeded GPP_b_ (Supporting Information Fig. 2), leading to negative NEM_b_ i.e., net heterotrophy throughout the duration of this study. The NEM_b_ rates at these two sites were similar and reached minimum values of −17.3 mmol m^−2^ d^−1^and −17.9 mmol m^−2^ d^−1^ during the spring, while maximum values of −89.8 mmol m^−2^ d^−1^ and −79.1 mmol m^−2^ d^−1^, were observed during the autumn (Fig. 5; Table 4).

## Discussion

### Water column vs. benthic metabolic activity

Due to higher concentrations of organic material and microbial densities in sediments compared to the overlying water (Tank et al. 2010), benthic activity is generally expected to dominate whole‐stream metabolism (Naegeli and Uehlinger 1997; Bott et al. 2006; Oliver and Merrick 2006). However, our results revealed that a substantial amount of respiration could occur in the water column, with ER_w_ up to 26.2 mmol m^−2^ d^−1^ (Table 3). Respiration in the water column was most important in the turbid water at the CL reach, where it represented 71.3%, on average, of the spring and summer whole‐stream respiration, ER_b+w_. On an annual basis, the water column metabolism still contributed to 28% of the mean ER_b+w_, at this site (Table 5). Conversely, ER_w_ at the CW reach, during the spring, was below the level of detection. On average, ER_w_ represented ∼ 28% of the annual ER_b+w_ across season and geology (Table 5). Primary production in the water column was particularly high during the spring campaign, where it accounted for > 90% of the GPP_b+w_, reaching as high as 290 mmol m^−2^ d^−1^ during a pronounced peak in algal primary production at the CL reach. While that appeared to be an extreme case for the upper catchment of the River Avon, it was well within the range for eutrophic lakes and rivers and peak spring production in headwaters (e.g., Likens 1975; Roberts et al. 2007).

**Table 5:** Relative benthic contributions to the total seasonal stream metabolism for CL, CW, and GN. The total rates of ER, GPP, and NEM are defined as the sum of benthic and water column contributions as listed in Tables 3, 4.

Campaign	Site	ER_b_/ER_b+w_ (%)	GPP_b_/GPP_b+w_ (%)	NEM_b_/NEM_b+w_ (%)
Spring	CL	72.3	7.5	7.3^b^
	CW	>99.9	>99.9	>99.9
	GN	86.8	42.3	38.0^b^
Summer	CL	28.7	‐a	54.7
	CW	88.5	85.7	82.7
	GN	77.2	‐a	54.3
Autumn	CL	94.0	‐a	91.7
	CW	93.2	>99.9	116.8^b^
	GN	87.3	‐a	83.2
Winter	CL	n.a.^c^	n.a.^c^	n.a.^c^
	CW	77.3	‐a	84.1
	GN	77.6	‐a	92.8

a Ratio could not be calculated (no production, *see* Table 4).

b Note that benthic and water column NEM values were of opposite sign.

c No data available (*see* Table 4). Water column incubation data for the winter campaign could not be collected due to catchment‐wide flooding.

Opposing net autotrophic and net heterotrophic activity for the benthic compartment and water column were occasionally observed. For example, during the spring, the benthic compartments at the CL and GN reaches were net heterotrophic (Table 4) while the water column, which mostly contributed to the system metabolism, was net autotrophic (Table 3). During those periods, the benthic compartment was likely mainly relying on an autochthonous carbon source, recycling part of the phototrophic biomass produced in the water column. Conversely, in the autumn at the CW reach, the benthic compartment was net autotrophic while the water column was dominated by heterotrophic activity, suggesting respiration in the water column was sustained by organic material supplied by streambed leakage and/or runoff. Potential effects of scouring on benthic biofilm during high flow regimes (e.g., Uehlinger 1991) cannot be excluded but such processes were never visually apparent here.

### Stream metabolism across geologies and areal upscaling

#### Seasonal variability

The dynamics in metabolic activity in the water column and benthic compartment were also reflected in the derived estimates of whole‐stream net ecosystem metabolism, NEM_b+w_. It should be noted that these dynamics were not constrained, or seasonally limited, by local nutrient availability as nutrient concentrations were generally high (Supporting Information Table 1). At the Chalk reach (CW), the system scale NEM_b+w_ was > 0 during the spring, summer, and autumn, indicating net autotrophic conditions (Fig. 5; Table 3). During the autumn, O_2_ concentration showed clear diel dynamics, but O_2_ availability was constantly below saturation, despite net autotrophy (Fig. 5; Table 2). That offset in O_2_ was attributed to increased hydraulic connectivity during the wet periods, autumn and winter, promoting inflow of O_2_ depleted water upstream of the CW reach (Heppell et al. unpubl.). During the winter, under similar wet conditions, primary production was negligible, resulting in dampened O_2_ dynamics, undersaturation in O_2_ (Table 2) and net heterotrophy (Fig. 5). The annual‐averaged daily stream metabolism at the CW reach, approximated as the average of each seasonal NEM_b+w_ rates, amounted to 31.9 mmol m^−2^ d^−1^ indicating that the system was net autotrophic on an annual timescale, producing, and potentially exporting, phototrophic biomass downstream.

For most of the year, the reach at CL was characterized by NEM_b+w_ < 0 (Table 4) which indicated net heterotrophy and an allochthonous source of organic carbon. Due to the flashiness of the site, there is close connectivity between the stream and the surrounding land, which facilitates the supply of relatively unprocessed terrestrially‐derived organic material (e.g., Battin et al. 2008). This was especially apparent during the wet autumn, when NEM_b+w_ was ≪ 0 at both CL and the adjacent GN reach (see below) (Fig. 5). Similarly, during the winter flooding, NEM_b+w_ was elevated, but to a lesser extent than during the autumn, suggesting that the system was still predominantly reliant on allochthonous organic carbon. The hydrological connectivity during that period was higher, with shorter terrestrial residence times than during the autumn (Heppell et al. 2017), indicating that the supplied organic material was of a more refractory nature. A peak in algal primary production induced transient net autotrophy during the spring campaign, but the site remained net heterotrophic on an annual basis (Tables 3, 4). The overall NEM_b+w_ amounted to −43.5 mmol m^−2^ d^−1^ (Fig. 5) and the area‐specific activity balanced the apparent net production of organic material at the CW reach.

At the Greensand reach (GN), spring NEM_b+w_ was > 0 (Fig. 5) as a result of net autotrophy in the water column offsetting the net heterotrophic benthic compartment (Tables 3, 4). However, the water column was net heterotrophic during the summer, leading to an overall net heterotrophic reach (Fig. 5). Similarly to the CL reach, the autumn and winter campaigns at the GN reach were characterized by NEM_b+w_ ≪ 0, which coincided with extensive flooding and import of organic carbon. Integrated over the year, the GN reach was net heterotrophic (approximately −38.4 mmol m^−2^ d^−1^; Fig. 5), with the stream activity presumably sustained mainly by allochthonous organic material.

About 82% of the upper catchment of the River Avon is dominated by Chalk and the net autotrophy of these areas largely outbalanced the net heterotrophy of the clay and Greensand dominated tributaries that only account for 18% of the area of the upper catchment. On an annual basis, therefore, the upper catchment is likely to be net autotrophic, representing a net source of organic material for export downstream and potentially all the way to the coast.

### Drivers of stream metabolism

#### Light

**Figure 6:**
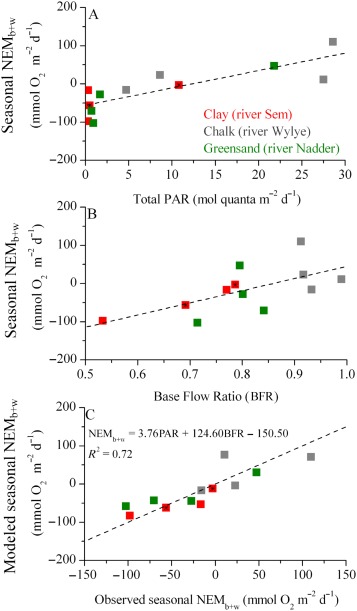
Stream net ecosystem metabolism (NEM_b+w_) over changing light availability and hydrology. (**A**) Seasonal NEM_b+w_, as a function of the average daily‐integrated PAR at the streambed (total PAR, in mol quanta m^−2^ d^−1^) during each seasonal campaign. The dashed line is a linear relationship (*R* ^2^ = 0.67). (**B**) Seasonal NEM_b+w_ as a function of the average ratio between base flow and stream discharge (BFR), calculated for 9 d of hydrological data during the respective seasonal campaigns. BFR values for spring 2013 were calculated for a hydrological comparable period in 2014, as prior data were unavailable. The dashed line represents the linear trend (*R* ^2^ = 0.42). (**C**) Relationship between the observed NEM_b+w_ obtained from AEC and incubation measurements and modeled NEM_b+w_ calculated as a function of the key drivers of stream metabolism, i.e., total PAR and BFR. The dashed line represents a 1 : 1 ratio. *, seasonal NEM_b+w_ at the CL for the whole‐spring period was obtained by weighting the spring campaign NEM_w_ to 1 week of peak primary production in the water column (*see* Results). The autumn NEM_w_ rate was used for the winter NEM_w_, as the latter could not be obtained in the field (*see* Table 3).

The NEM_b+w_ rates exhibited a clear relationship with the average daily integral of PAR at the streambed (Fig. 6A), which indicated a shift between net heterotrophy to net autotrophy at light levels above 12 mol quanta m^−2^ d^−1^. All sites complied to a unifying general trend, despite the fact that light availability also incorporates seasonal trends (e.g., daytime duration, overall solar irradiance). Light availability, however, is further influenced by stream turbidity levels, which in turn are modulated by hydrology. For example, short‐lived flashy flows lasting up to several days can transiently enhance turbidity and limit GPP (e.g., Leggieri et al. 2013). On annual time scales, stream turbidity is modulated by sub‐catchment geologies, with reduced turbidity at the reaches fed by groundwater and higher turbidity at the reaches dominated by surface runoff (e.g., Fig. 1E–G).

#### Hydrology

The reduced amount of sites investigated in this study limits a general assessment of the sub‐catchment geologies. However, as shown by Heppell et al. (2017), these sites fall within a gradient of BFI (Supporting Information Fig. 4) and thus of hydrological connectivity, which again is expected to relate to the biological and biogeochemical function of the tributaries. The degree of hydrological connectivity is typically expressed as the ratio between base flow^4^ and stream discharge, which represents the proportion of river runoff derived from stored sources due to catchment permeability (e.g., Gustard et al. 1992). Over the long‐term integral (annual to decadal) of BFI, the ratio reflects the permeability of the underlying catchment geology, while over months it reflects seasonal features, e.g., wet periods during the autumn and winter. The ratio also varies on a daily to weekly basis following concurrent dynamics in the local precipitation and surface runoff (e.g., event BFI; Outram et al. 2016). Because NEM_b+w_ rates exhibit strong seasonal dynamics (Fig. 5), large error bars may mask any potential functional relationships between stream metabolism and BFI. Thus, to relate NEM_b+w_ to the hydrological connectivity within the sub‐catchments, it is more appropriate to use the Base Flow Ratio (BFR) as expressed on a timescale comparable to the dominant biological responses (e.g., the timescale at which the majority of organic carbon is being over‐turned). We found that BFR values computed over a period of 9 d (*n* = 9), starting before each field campaign and including the campaigns, revealed a statistically robust relationship to NEM_b+w_ (Fig. 6B). Reducing the period to only a few days, or extending it to several weeks or a month, weakened the relationship. This indicated that, on average, BFR dynamics are imprinting the reach scale carbon turnover at a time scale of 9 d. This includes hydrological driven entrapment and retention of allochthonous and autochthonous organic material being mineralized within the reach, as well as responses in primary productivity. The imprint might therefore vary in time as a result of seasonal changes in the supply of organic carbon, from allochthonous and/or autochthonous sources, and changes in stream morphology‐ and hydrology‐driven residence time within the reach. For example, during months with extensive aquatic vegetation cover, the imprint of allochthonous organic matter in vegetated reaches is expected to last longer than in nonvegetated reaches of similar hydrology, due to the vegetation reducing flow velocity and promoting the settling and entrapment of the allochthonous material (e.g., Cotton et al. 2006; Jones et al. 2012). In contrast, during the winter period, reach‐scale carbon turnover will likely occur at comparable time scales in both vegetated and nonvegetated reaches. On an annual basis, we found that 9 d represented an average between those seasonal and hydrological variabilities within and across the sub‐catchments.

The BFR dynamics do not only modulate the transport and cycling of organic material but also the concurrent primary production of the reach. This was particularly evident during rain events, when surface runoff resulted in an increase in water turbidity and strongly reduced primary production (e.g., GN reach in autumn; Supporting Information Fig. 2). The BFR therefore represents an integral predictor for the metabolic activity at the reach scale, including complex system responses toward hydrological characteristics and seasonal dynamics (e.g., Outram et al. 2016).

Seasonal NEM_b+w_ ranged from generally net heterotrophic at the clay reach, characterized by a low average BFR (0.53–0.79), to mainly net heterotrophic in the Greensand, with an intermediary BFR (0.71–0.84), to predominately net autotrophy in the Chalk with a high BFR (> 0.91). BFR values were the lowest during the wet autumn campaign at the CL and GN reaches (Fig. 6B), suggesting elevated contributions from rain event driven surface runoff and shallow throughflow, and generally coincided with periods of heterotrophy at the respective reaches. Overall, we found a general shift from net heterotropy at low BFR, with variable hydrography and higher surface runoff, and rapid throughflow, to net autotrophic conditions at the highly permeable, groundwater‐feed Chalk sub‐catchments (high BFR). This functional trend appeared consistent across seasons and sub‐catchment geologies.

Riverine NEM thus expresses a close relationship to both light availability and the BFR, and, combining these two drivers into one overall relationship improved the overall fit to the data (Fig. 6). Such novel relationships could provide a powerful tool for upscaling local carbon dynamics to catchment and regional scales, but could also facilitate qualitative and quantitative projections of riverine responses toward anthropogenic and climate‐induced changes. It remains to be seen to what extent similar relationship can be applied to catchments across a broader spectrum of land uses, and whether it may be representative for headwater tributaries or temperate lowland rivers in general which would make our approach a valuable tool for riverine management.

### Benthic oxygen exchange as quantified by AEC in rivers

Stream metabolism has most often been quantified as whole‐stream metabolism, which includes contributions from the benthic compartment and the water column, by “open‐system” methods (Marzolf et al. 1994; Mulholland et al. 2001; Izagirre et al. 2008; Demars et al. 2015). Assessments of benthic metabolism have been confined to invasive chamber incubations at patch scales of less than 0.1 m^2^ (e.g., Hickey 1988; Bott et al. 1997). Upscaling chamber measurements demands multiple deployments and also requires several assumptions about the relative contribution of each patch (Glud and Blackburn 2002; Young et al. 2008) and considerations about the potential bias in light availability and hydrodynamics inside the chamber enclosure (e.g., Dodds and Brock 1998; Cook et al. 2007; Berg et al. 2013).

The AEC technique, enables noninvasive measurement of oxygen exchange over 10s – 100s m^2^ benthic area under unaltered light and hydrodynamic conditions (Berg et al. 2003). The AEC technique thus effectively covers the reach scale and can be applied over hard substrata, where traditional benthic techniques cannot. While the technique has previously been successfully applied to investigate benthic oxygen exchange in rivers (Chipman et al. 2012; Berg et al. 2013; Murniati et al. 2015) and impounded riverine systems (McGinnis et al. 2008; Lorke et al. 2012), it has only very recently been applied to assess seasonal stream metabolism (Koopmans and Berg 2015). General considerations on AEC applications have been extensively evaluated in recent publications (e.g., Lorrai et al. 2010; Berg et al. 2013; Holtappels et al. 2013; Berg et al. 2015; Donis et al. 2015; see also Methods in this study). However, there are aspects that are of particular relevance for deployments in headwaters and lower order streams that need further consideration.

#### AEC footprint

One main advantage of the AEC approach is the noninvasive interrogation of a relatively large area of the streambed, i.e., the AEC footprint, which can be estimated as a function of *h* and *z*_0_ (Berg et al. 2007; *see* Methods). As the estimates are independent from discharge, they can be easily extrapolated across seasonal hydrographs. For the present study, the AEC footprints encompassed a streambed area of 9–184 m^2^ (on average ∼ 60 m^2^). The AEC also integrates the contributions from heterogeneous and patchy habitats, though the resolvable patchiness is site‐specific. This can be quantified as the largest square‐sized area of heterogeneous benthic surface fully integrated within the footprint, from specific *h* and *z*_0_ (*see* Rheuban and Berg 2013). The resolvable patch sizes were, on average, 0.8 × 0.8 m at CW, and 1.1 × 1.1 m at the GN and CL reaches, respectively. These patch sizes were markedly larger than the visually observed physical patch variations at each site, indicating that habitat patchiness was generally well integrated in our measurements. Flux contributions within the footprint are also not homogenous. Following Berg et al. (2007), we calculated that the largest contributions to the benthic flux were located in a region 1–5 m upstream of the instrument, termed the region of maximum flux, while the contributions further decreased near‐exponentially with increasing distance from the instrument. One challenge for riverine AEC is therefore to include site‐specific features, such as lotic vegetation, in the footprint while ensuring an unobstructed flow field, to avoid secondary flow phenomena and flux inhomogeneity within the water column.

#### Lotic vegetation

The occurrence of lotic vegetation was minimal at CL and scattered as isolated macrophytes patches at GN; benthic primary production was mainly mediated by benthic microalgae. In contrast, CW was characterized by the frequent occurrence of small (0.5 × 0.5 m) macrophyte patches, which were included and well‐integrated in the footprint, and by sparse, larger patches (Fig. 1B). Those large patches exceeded *h* and were deliberately left outside the footprint area to avoid flow disturbances. Thus, part of the overall metabolic contribution from macrophytes might not have been fully integrated in the AEC approach as applied at CW. Contributions from lotic vegetation are usually poorly defined in assessments of stream metabolism. Emergent aquatic plants typically do not contribute significantly to stream metabolism, as most of the gas exchange is mediated via the atmosphere (e.g., Caraco et al. 2006). Also, net primary production of riverine submerged macrophytes generally contributes little to system net production (King and Ball 1966; Fisher and Carpenter 1976; Hill and Webster 1982, 1983). This is mainly due to concurrent turnover of entrapped highly reactive material underneath the patches (Sand‐Jensen, 1998; Cotton et al. 2006). Trimmer et al. (2009) showed that respiration rates associated with such macrophyte patches ranged between 17 mmol m^−2^ d^−1^ and 41 mmol m^−2^ d^−1^, peaking during the summertime. Thus, although macrophytes are contributing to the stream GPP and ER rates, those contributions are likely comparable in magnitude. Therefore, the contribution to NEM of the few large patches of macrophytes, which were excluded from the AEC footprint, was presumably small compared to the contribution from the benthic compartment in general.

#### Groundwater

The inflow of O_2_‐depleted groundwater into streams with permeable sediments can affect riverine O_2_ dynamics and assessment of whole‐stream metabolism (Mulholland et al. 2001; McCutchan et al. 2002; Hall and Tank 2005; Koopmans and Berg 2015). For example, Koopmans and Berg (2015) reported that anoxic water reaching the streambed during high groundwater inflow rates (0.47 m d^−1^) might generate oxygen fluxes equivalent to −126 mmol m^−2^ d^−1^ to −144 mmol m^−2^ d^−1^, representing ∼ 40% of the whole‐stream ER. In our study, phases of high groundwater inflow, e.g., wet periods in autumn and winter, occasionally lowered the background O_2_ concentration in the water column throughout the sub‐catchment. However, the maximum groundwater‐induced contribution to the local O_2_ consumptions at the studied reaches was overall < 1 mmol m^−2^ d^−1^, being on average < 3% of the biological benthic O_2_ consumption, and therefore negligible in the present context.

Provided appropriate consideration of the issues discussed above is made, AEC measurements allow robust assessment of reach‐scale streambed metabolism in rivers. AEC measurement can thus complement open‐system assessment (e.g., Koopmans and Berg 2015) to provide a better insight into the metabolic dynamics within the different riverine compartments. The noninvasive nature of the technique and its large spatial integration also provide a powerful tool to investigate benthic dynamics in stream metabolism across biomes at the reach scale and potentially for the upscaling of metabolic trends to catchment‐wide assessment of riverine contributions to the regional cycling of organic material.

## Supporting information

Supporting Information

## Footnotes

1 River Avon catchment upstream of 50° 58' 28.235'' N / 1° 45' 15.233'' W; data available at http://www.ceh.ac.uk/data/nrfa

2 Reach designations within the Macronutrient Cycles programme project were CW2, GN1, and AS2 (for CL).

3 River Wylye flow were obtained from the Environment Agency gauging station at Brixton Deverill, located at the upstream end of the CW reach (Station ID: 3322).

4 The base flow was determined from daily time series of discharge following the smoothing and separation method of Gustard et al. (1992). See Supporting Information for further details on BFI and BFR (Supporting Information Fig. 4).
