# Supplementary material for: Reach‐scale river metabolism across contrasting sub‐catchment geologies: Effect of light and hydrology
Source: Limnol Oceanogr. 2017 Jul 5;62(Suppl Suppl 1):S381–99. doi: 10.1002/lno.10619 (PMC5724700; doi:10.1002/lno.10619)
Supplement: Supplementary file 1 — Supporting Information [file LNO-62-S381-s001.docx]

**Supplemental Information**

SI Table 1. Overview of average inorganic nutrient concentrations during each field campaign at clay river Sem (CL), Greensand river Nadder (GN) and Chalk river Wylye (CW). Data are presented as mean ± standard deviation (number of samples).

| Campaign | Site | Ammonium  (µmol L^-1^) | Phosphate  (µmol L^-1^) | Nitrate  (µmol L^-1^) | Nitrite  (µmol L^-1^) |
| --- | --- | --- | --- | --- | --- |
| Spring | CL | 1.9 ± 0.3 (2) | 0.5 ± 0.1 (2) | 116.0 ± 2.0 (2) | 0.4 ± 0.5 (2) |
|  | CW | 0.6 ± 0.2 (2) | 0.8 ± 0.2 (2) | 480.9 ± 21.3 (2) | 0.6 ± 0.1 (2) |
|  | GN | 1.0 ± 0.6 (2) | 1.1 ± 0.6 (2) | 304.8 ± 51.1 (2) | 0.7 ± 0.1 (2) |
| Summer | CL | 3.6 ± 2.7 (44) | 4.4 ± 1.9 (44) | 58.5 ± 40.5 (44) | 0.7 ± 0.3 (44) |
|  | CW | 5.9 ± 5.0 (6) | 0.9 ± 0.3 (6) | 415.0 ± 58.2 (6) | 1.0 ± 0.3 (6) |
|  | GN | 3.8 ± 4.6 (6) | 2.2 ± 0.6 (6) | 274.1 ± 51.9 (6) | 0.8 ± 0.3 (6) |
| Autumn | CL | 7.6 ± 1.7 (26) | 1.0 ± 0.3 (26) | 260.7 ± 63.7 (26) | 0.6 ± 0.1 (26) |
|  | CW | 3.1 ± 1.1 (4) | 1.0 ± 0.2 (4) | 405.1 ± 25.3 (4) | 1.2 ± 0.1 (4) |
|  | GN | 8.8 ± 2.1 (2) | 1.0 ± 0.1 (2) | 218.6 ± 58.2 (2) | 0.7 ±0.2 (2) |
| Winter | CL | 10.6 ± 1.2 (29) | 2.4 ± 0.5 (29) | 94.9 ± 11.2 (29) | 0.5 ± 0.1 (29) |
|  | CW | 0.9 ± 0.4 (5) | 1.1 ± 0.2 (5) | 424.1 ± 51.9 (5) | 0.2 ± 0.0 (5) |
|  | GN | 8.2 ± 6.2 (6) | 2.1 ± 0.3 (6) | 301.9 ± 49.8 (6) | 0.7 ± 0.2 (6) |


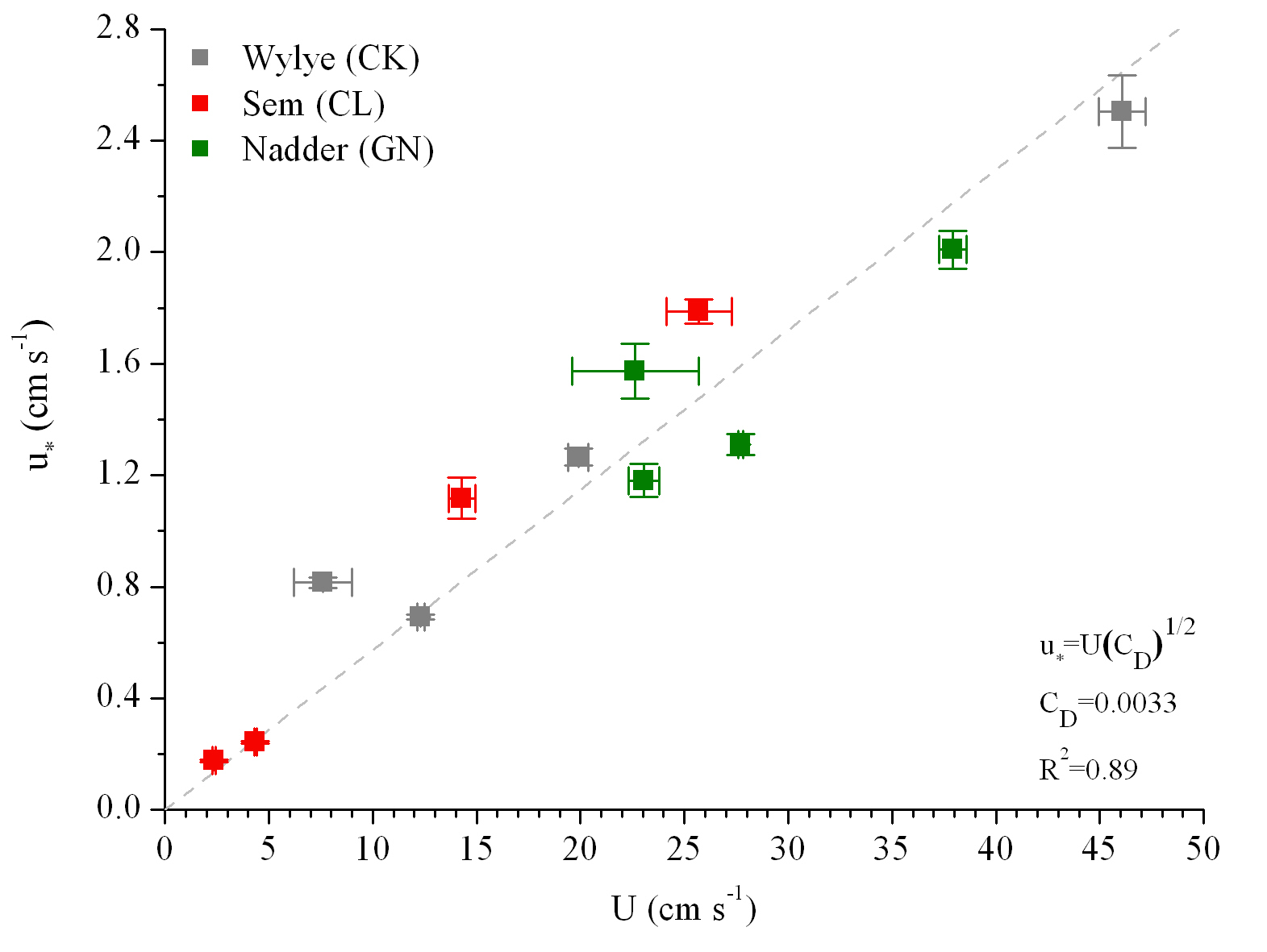


SI Figure 1. Flow dependency of friction velocity (u_*_) within the sub-catchment reaches. Shown are the seasonal averages for u_*_ as a function of mean flow velocity (U) for the clay river Sem (CL, triangles), Chalk river Wylye (CW, squares) and Greensand river Nadder (GN, stars). The dashed line indicates the best linear fit, *u*_*_ = *U*(*C*_D_)^1/2^, with C_D_ being the bottom drag coefficient. Note that the highest flow at each site was observed during a 2014 flooding event.





SI Figure 2. Seasonal gross primary production (*GPP*) and ecosystem respiration (*ER*) rates in the water column and benthic compartment. A) Seasonal rates of *ER* (black bars) and *GPP* (grey bars) in the water column (w) and benthic compartment (b) for clay river Sem (CL). B) Seasonal *ER* rates and *GPP* rates (red bars) for Chalk river Wylye (CW). C) Seasonal *ER* rates and *GPP* rates (green bars) for Greensand river Nadder (GN). Water column rates are based on bottle incubations while benthic rates were obtained using the non-invasive Aquatic Eddy Covariance Technique. Water column incubation data for the winter campaign at CL could no be collected due to flooding.


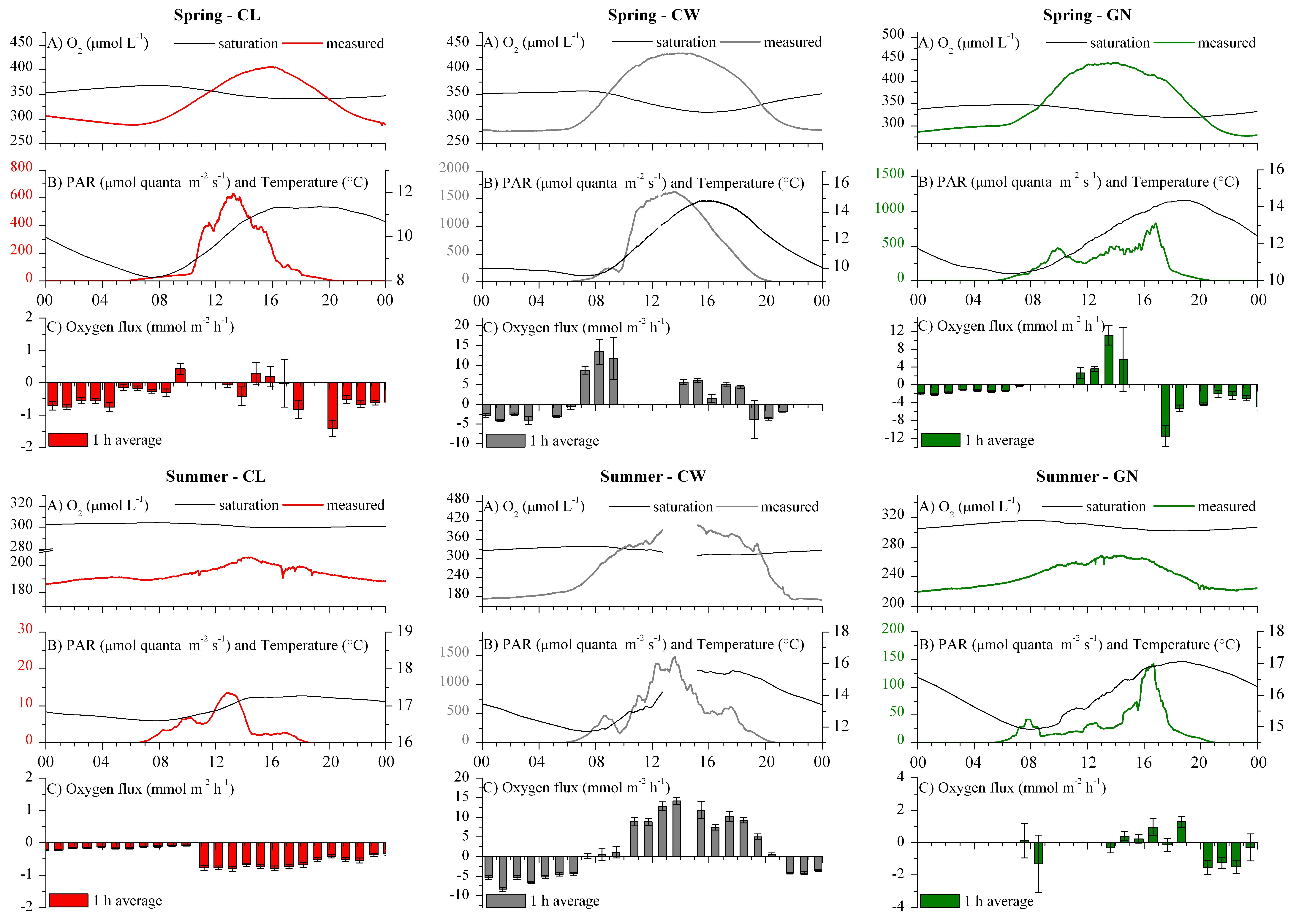
SI Figure 3. Typical AEC oxygen fluxes and auxiliary time series data during the spring (top boxes) and summer (bottom boxes) at clay river Sem (CL, left), Chalk river Wylye (CW, center), and Greensand river Nadder (GN, right). Each box represents a 24 h selection of (A) in situ near-riverbed absolute O_2_ concentration (red, blue or green lines) and O_2_ concentration at saturation (black lines), streambed PAR in µmol quanta m^-2^ s^-1^ (red, blue or green lines) and water column temperature (black line), and (C) turbulent AEC-based oxygen fluxes with standard error. Time series of PAR were averaged to 1 h to highlight the dominant trends.

SI Figure 4. Example of stream discharge and base flow time series for the river Wylye (top), river Nadder (center) and river Sem (bottom). Base flow and base flow index (*BFI*) were obtained using the smoothing and separation method of Gustard et al. (1992). *BFR*_n_ represents the ratio between base flow and discharge and is computed over *n* days (not shown). For further details on the *BFI* for these rivers see Heppell et al. (2017).
